# Peripheral blood inflammatory indices across thyroid functional states in Graves’ disease: associations with liver biochemical abnormalities and hyperthyroid relapse

**DOI:** 10.3389/fendo.2026.1863070

**Published:** 2026-08-21

**Authors:** Wenjie Sun, Hui Chen

**Affiliations:** 1 The Second Clinical Medical College of Lanzhou University, Lanzhou, Gansu, China; 2 Department of Endocrinology and Metabolism, Lanzhou University Second Hospital, Lanzhou, Gansu, China

**Keywords:** Graves’ disease, hyperthyroid relapse, liver biochemical abnormalities, monocyte-to-lymphocyte ratio, pan-immune-inflammation value, peripheral blood inflammatory indices, thyroid function

## Abstract

**Background:**

Graves’ disease is an organ-specific autoimmune disorder driven by thyrotropin receptor antibody (TRAb) and accompanied by persistent immune and inflammatory activation. Peripheral blood inflammatory indices are readily available and have been increasingly used to assess inflammation in autoimmune diseases. However, their clinical significance in Graves’ disease across different thyroid functional states is unknown.

**Methods:**

In this retrospective study, 687 patients with Graves’ disease were enrolled. Five peripheral blood inflammatory indices were calculated, including the neutrophil-to-lymphocyte ratio (NLR), monocyte-to-lymphocyte ratio (MLR), systemic immune-inflammation index (SII), systemic inflammation response index (SIRI), and pan-immune-inflammation value (PIV). Patients were stratified and compared according to thyroid functional status. Because sex distribution differed among the groups, sex-adjusted median regression models were additionally fitted, and group-by-sex interactions were examined. Correlation analysis, logistic regression, and receiver operating characteristic (ROC) curve analysis were used to evaluate associations of these indices with clinical characteristics, liver biochemical abnormalities, and hyperthyroid relapse. The outcome models were internally validated using bootstrap resampling and repeated 10-fold cross-validation.

**Results:**

Sex distribution differed significantly among the thyroid functional groups. After adjustment for sex, MLR, SIRI, and PIV remained significantly higher in the overt hyperthyroid group than in the subclinical hyperthyroid and euthyroid groups. In patients with overt hyperthyroidism, MLR was positively correlated with age, FT3, FT4, ALT, and AST (r_s_ = 0.152–0.206, all *P* < 0.05). Multivariable logistic regression identified TRAb, hemoglobin, and standardized MLR as independent factors associated with liver biochemical abnormalities, and the resulting model showed limited-to-moderate discrimination (apparent AUC, 0.703; optimism-corrected AUC, 0.692; cross-validated AUC, 0.680). Among the five inflammatory indices, PIV showed the highest individual discrimination for hyperthyroid relapse (AUC, 0.734). The TRAb–PIV model showed relatively good internal discrimination (apparent AUC, 0.815; optimism-corrected AUC, 0.805; cross-validated AUC, 0.788).

**Conclusion:**

Compared with NLR and SII, the monocyte-containing indices MLR, SIRI, and PIV showed clearer differences across thyroid functional states in Graves’ disease and may provide complementary inflammation-related information. Among them, MLR was associated with liver biochemical abnormalities, whereas PIV showed potential adjunctive value for hyperthyroid relapse risk assessment.

## 1 Introduction

Hyperthyroidism is a hypermetabolic disorder caused by excessive production and secretion of thyroid hormones, of which Graves’ disease (GD) is the most common etiology. GD is an organ-specific autoimmune thyroid disease caused by thyrotropin receptor antibody (TRAb)-mediated activation of the thyroid-stimulating hormone (TSH) receptor, leading to excessive thyroid hormone secretion. It is associated with breakdown of immune tolerance and inflammatory responses mediated by T cells, B cells, and multiple cytokines (1–3). Previous work has shown that interferon-γ-mediated Th1-type cellular immune responses are present in GD (4–6), accompanied by chronic low-grade inflammation (6). However, in addition to inflammation induced by autoimmune dysregulation, excessive thyroid hormone may also contribute to inflammation (4, 7–10). Thus, it may be hypothesized that immune imbalance and thyroid hormone affect the inflammatory microenvironment of GD, which may contribute to disease initiation and progression (10, 11).

Recently, inflammation-related indices derived from routine peripheral blood parameters have attracted increasing attention due to their convenience, low cost, and reproducibility. The neutrophil-to-lymphocyte ratio (NLR) and monocyte-to-lymphocyte ratio (MLR) are the most common and widely used inflammatory markers and have shown potential diagnostic or prognostic value in inflammatory diseases and malignancies (12, 13). Building on these simple ratios, more comprehensive composite indices have been developed, including the systemic immune-inflammation index (SII) (14), systemic inflammation response index (SIRI) (15), and pan-immune-inflammation value (PIV) (16). Neutrophils and monocytes primarily reflect innate immune activation and proinflammatory activity, whereas lymphocytes reflect adaptive immune status (17). Platelets also contribute to inflammation and immune regulation through direct interactions with neutrophils, monocytes, and lymphocytes and by releasing soluble mediators that modulate leukocyte activation, recruitment, and immune responses (18). Accordingly, SII is intended to capture neutrophil- and platelet-related inflammatory activity relative to lymphocyte status, SIRI incorporates both neutrophil- and monocyte-related innate immune activity, and PIV integrates all four cellular components. By combining different aspects of innate immunity, adaptive immunity, and platelet-mediated inflammation, these composite indices may provide a more comprehensive representation of systemic inflammatory and immune status than individual cell counts or simple cell-count ratios. Because they can be calculated directly from routine complete blood counts without additional testing, these indices were selected as practical candidate biomarkers for evaluating systemic inflammatory status and exploratory risk stratification in patients with Graves’ disease.

Existing studies in GD have focused primarily on NLR, MLR, and SII. In particular, several studies have reported comparable NLR levels in patients with GD and healthy controls, or even lower NLR levels in patients with GD (19–21), whereas higher NLR levels in Graves’ orbitopathy (GO) may be associated with disease activity (22, 23). In addition, patients with GO and overt hyperthyroidism had higher NLR, MLR, and SII than euthyroid patients with GO, suggesting that thyroid functional status may influence these indices (24).

However, important limitations remain in the existing literature. First, SIRI and PIV have received relatively little attention in GD. Second, previous studies have generally stratified patients with GD according to treatment status, most commonly comparing newly diagnosed untreated patients with those who achieved euthyroidism after treatment. Such treatment-based classification may exclude patients with subclinical hyperthyroidism and therefore cannot fully characterize peripheral blood inflammatory indices across the spectrum of thyroid functional states. Whether the inflammatory profile of subclinical hyperthyroidism more closely resembles that of overt hyperthyroidism or euthyroidism has therefore not been established. Third, although these indices have shown diagnostic and prognostic value in various autoimmune diseases (25–27), their associations with clinically relevant outcomes in GD are poorly defined. Liver biochemical abnormalities are common in GD (28), and hyperthyroid relapse after ATD withdrawal remains a major challenge (2), but whether peripheral blood inflammatory indices provide additional information regarding either outcome is unknown.

To address these knowledge gaps, we systematically compared NLR, MLR, SII, SIRI, and PIV among patients with GD and overt hyperthyroidism, subclinical hyperthyroidism, or euthyroidism. We further investigated their associations with liver biochemical abnormalities in patients with overt hyperthyroidism and evaluated their discriminatory performance for hyperthyroid relapse occurring within 12 months after the index assessment among patients who were off ATD therapy and biochemically euthyroid at that assessment. This study aimed to explore the potential adjunctive value of these indices for assessing inflammatory status and supporting exploratory risk stratification in GD.

## 2 Methods

### 2.1 Study design and setting

This retrospective observational study was conducted in the outpatient clinic of Lanzhou University Second Hospital from October 2024 to March 2025. Consecutive patients were screened for eligibility during the study period. The study protocol was approved by the Ethics Committee of the Second Hospital of Lanzhou University (approval no. 2024A-389) and followed the principles of the Declaration of Helsinki.

### 2.2 Participants

The diagnosis of Graves’ disease was established in accordance with the 2018 European Thyroid Association Guideline for the Management of Graves’ Hyperthyroidism (29). For newly diagnosed patients, Graves’ hyperthyroidism was diagnosed based on biochemical hyperthyroidism, defined as suppressed TSH accompanied by elevated FT4 and/or FT3 concentrations, together with etiological evidence supporting GD, primarily a positive TRAb result. Characteristic thyroid ultrasonographic findings, including diffuse hypoechogenicity and increased vascularity, were considered supportive evidence. In diagnostically challenging cases, thyroid scintigraphy was additionally performed to clarify the etiology of hyperthyroidism. For patients with subclinical hyperthyroidism or biochemical euthyroidism at the index assessment, eligibility required a documented previous diagnosis of GD supported by historical evidence of biochemical hyperthyroidism, together with previously documented positive TRAb results and/or characteristic thyroid imaging findings.

Patients were included if they met all of the following criteria: (1) a documented diagnosis of GD established according to the criteria described above; (2) their first outpatient visit satisfying all study eligibility requirements occurred between October 2024 and March 2025; (3) they underwent concurrent complete blood count and thyroid function testing at the index visit; and (4) they had available TRAb results and essential clinical data. Patients were excluded if they met any of the following criteria: (1) hyperthyroidism due to causes other than GD; (2) concomitant autoimmune diseases other than GD; (3) active infection; (4) malignancy; (5) hematologic disease; (6) chronic liver disease; (7) use of glucocorticoids or immunosuppressive agents within 1 month before the index visit; or (8) missing essential clinical or laboratory data, including thyroid function tests, TRAb, or complete blood count data.

A total of 687 patients with GD were included. Depending on the thyroid functional status at the index assessment, the population was stratified into three groups: overt hyperthyroidism (OH, n = 283), subclinical hyperthyroidism (SCH, n = 137), and euthyroidism (EU, n = 267). According to disease phase and antithyroid drug (ATD) treatment status at the index assessment, patients in the OH group were further classified into the initial-presentation overt hyperthyroidism subgroup (OH-IP, n = 54; newly diagnosed GD without previous ATD exposure), the overt hyperthyroidism during ATD treatment subgroup (OH-ATD, n = 191; previously diagnosed GD with overt hyperthyroidism while receiving regular ATD treatment), and the recurrent overt hyperthyroidism subgroup (OH-RP, n = 38; recurrent overt hyperthyroidism after ATD withdrawal following remission). Patients in the EU group were further classified into the euthyroidism during ATD treatment subgroup (EU-ATD, n = 170) and the euthyroidism after ATD withdrawal subgroup (EU-WD, n = 97). Subgroup classification was based on documented disease history, treatment records, and concurrent laboratory findings.

**Figure 1:**
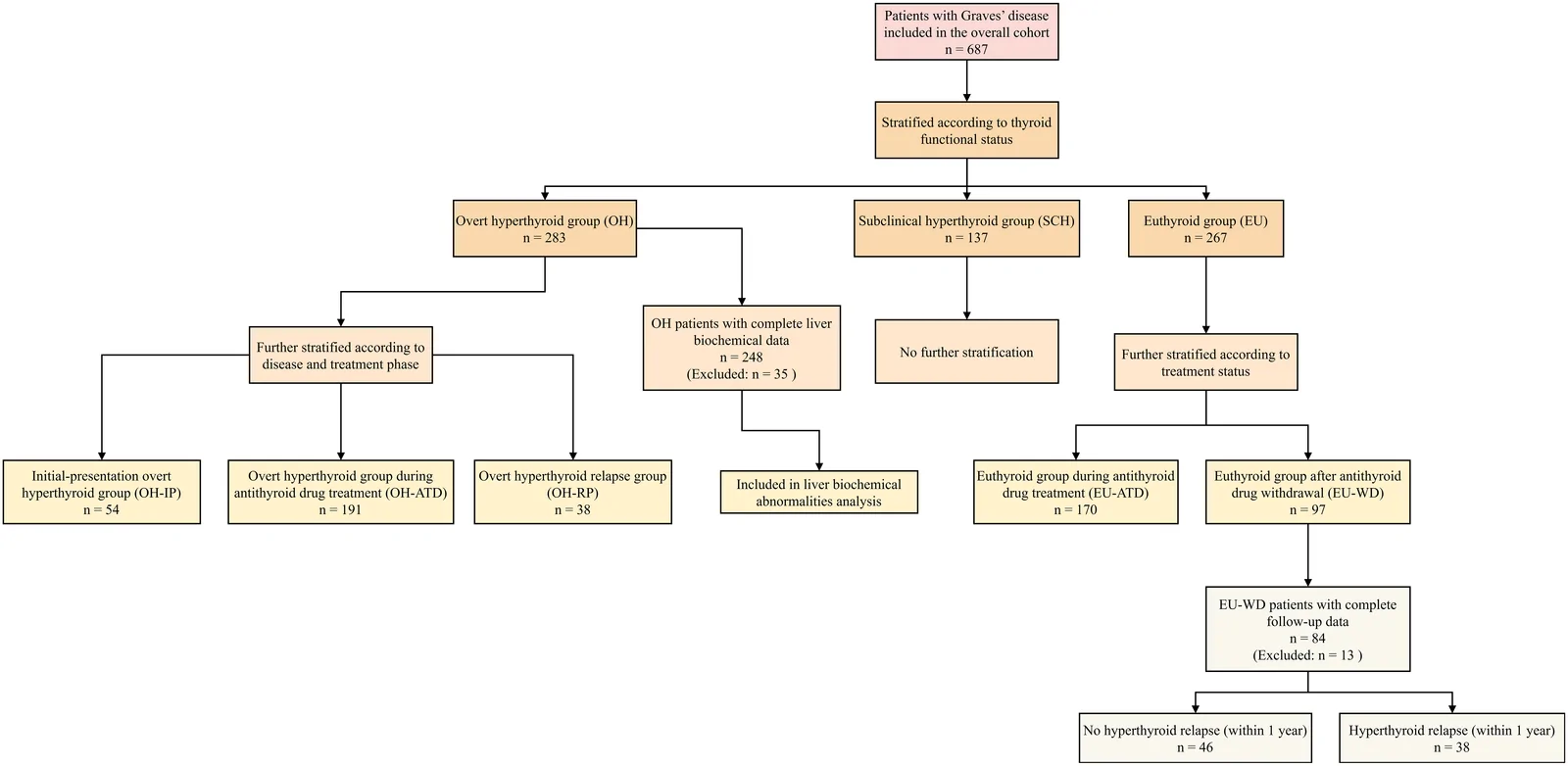
Flowchart of patient inclusion, thyroid functional stratification, subgroup classification, and derivation of the analytic cohorts for liver biochemical abnormalities and 12-month hyperthyroid relapse.

Because the analytic populations differed according to the study objectives, specific subgroups were defined for the outcome analyses. Analyses of liver biochemical abnormalities were restricted to patients in the OH group with complete liver biochemical data (n = 248). The hyperthyroid relapse analysis included 84 patients in the EU-WD group with complete 12-month follow-up data. During the 12-month follow-up from the index date, 38 of these 84 patients experienced hyperthyroid relapse. A flowchart showing patient inclusion, thyroid function-based stratification, subgroup classification, and the outcome-specific cohorts for the liver biochemical abnormalities and hyperthyroid relapse analyses is presented in **Figure 1**.

### 2.3 Clinical and laboratory data

Demographic characteristics (age and sex), thyroid function parameters, including total triiodothyronine (TT3), total thyroxine (TT4), free triiodothyronine (FT3), free thyroxine (FT4), thyroid-stimulating hormone (TSH), and thyrotropin receptor antibody (TRAb), liver biochemical parameters, including alanine aminotransferase (ALT), aspartate aminotransferase (AST), total bilirubin (TBIL), direct bilirubin (DBIL), and indirect bilirubin (IBIL), and complete blood count (CBC) parameters were recorded for all participants at the first visit. CBC parameters included white blood cell count (WBC), hemoglobin (Hb), neutrophil count (NEUT), lymphocyte count (LYM), monocyte count (MONO), and platelet count (PLT). Platelet indices included platelet distribution width (PDW), plateletcrit (PCT), platelet-large cell ratio (P-LCR), and mean platelet volume (MPV). Complete blood counts and differential leukocyte counts were measured using an XT-1800i automated hematology analyzer (Sysmex Corporation, Kobe, Japan). Leukocyte differential counts were determined using fluorescence flow cytometry, hemoglobin concentrations using a cyanide-free colorimetric method, and platelet counts using the electrical impedance method. Serum ALT, AST, TBIL, and DBIL concentrations were measured using an AU5800 automated biochemical analyzer (Beckman Coulter, Inc., Brea, CA, USA). ALT and AST were determined using kinetic enzymatic methods, whereas TBIL and DBIL were measured using automated colorimetric methods. IBIL was calculated as TBIL minus DBIL.

Serum TT3, TT4, FT3, FT4, and TSH concentrations were measured using an ADVIA Centaur XP automated immunoassay analyzer (Siemens Healthineers, Erlangen, Germany) with the corresponding reagent kits supplied by Siemens Healthcare Diagnostics Inc. (Tarrytown, NY, USA), based on direct chemiluminescent immunoassays. Serum TRAb concentrations were measured using a MAGLUMI X8 automated chemiluminescence immunoassay analyzer (Shenzhen New Industries Biomedical Engineering Co., Ltd., Shenzhen, China) with the corresponding MAGLUMI reagent kits supplied by the manufacturer, based on a magnetic microparticle chemiluminescence immunoassay. All laboratory measurements were performed in the central clinical laboratory of Lanzhou University Second Hospital in accordance with the manufacturers’ instructions and routine internal quality-control procedures.

Treatment status at the index assessment was obtained from contemporaneous outpatient records. Exact disease duration, cumulative ATD treatment duration, and actual ATD dose at blood sampling were incompletely documented and were therefore not included in the analyses.

Based on CBC data, five inflammation-related indices were calculated for each participant: NLR = NEUT/LYM; MLR = MONO/LYM; SII = PLT × NEUT/LYM; SIRI = NEUT × MONO/LYM; and PIV = PLT × NEUT × MONO/LYM.

### 2.4 Definitions of clinical variables

Thyroid functional classification was based on serum FT3, FT4, and TSH concentrations. The laboratory reference ranges were 2.77–6.31 pmol/L for FT3, 10.44–24.38 pmol/L for FT4, and 0.38–4.34 μIU/mL for TSH. Euthyroidism was defined as FT3, FT4, and TSH concentrations within their respective reference ranges. Overt hyperthyroidism was defined as TSH below the lower limit of the reference range, accompanied by FT4 and/or FT3 above the corresponding upper limit. Subclinical hyperthyroidism was defined as TSH below the lower limit of the reference range, with FT3 and FT4 concentrations within their respective reference ranges. TT3, TT4, and TRAb were recorded as additional descriptive variables but were not used for thyroid functional classification; the reference ranges for all thyroid-related parameters are provided in **Supplementary Table 1**.

Based on a previous systematic review and meta-analysis (28), liver biochemical abnormalities were operationally defined as an elevation above the laboratory-specific upper limit of normal in at least one of the following parameters: alanine aminotransferase (ALT) > 40 U/L, aspartate aminotransferase (AST) > 35 U/L, total bilirubin (TBIL) > 21 μmol/L, direct bilirubin (DBIL) > 4 μmol/L, or indirect bilirubin (IBIL) > 17 μmol/L. Patients with at least one abnormal parameter were classified as having liver biochemical abnormalities.

The index date was defined as the first eligible outpatient visit at which complete blood count and thyroid function tests were performed concurrently. Consistent with a previous study that used a comparable assessment during biochemical euthyroidism as the start of follow-up (30), patients were followed for 12 months from the index date. In the EU-WD cohort, hyperthyroid relapse was defined, based on a previously reported definition (31), as the first occurrence within 12 months after the index assessment of suppressed TSH accompanied by elevated FT4 and/or FT3 levels requiring reinitiation of ATD therapy in patients who were off ATD therapy and biochemically euthyroid at baseline. Hyperthyroid relapse outcomes were retrospectively ascertained through review of longitudinal outpatient clinical and laboratory records.

### 2.5 Statistical analysis

Data analysis was conducted with SPSS version 27 and R version 4.6.1. The distribution of continuous variables was examined before further analysis. Normally distributed data were reported as mean ± standard deviation (SD), and comparisons among multiple groups were performed using one-way analysis of variance (ANOVA). Non-normally distributed data were presented as median (interquartile range, IQR). Differences among multiple groups were analyzed using the Kruskal–Wallis H test, followed by Bonferroni-adjusted pairwise comparisons when the overall test was significant. Categorical variables were reported as number (percentage), and comparisons between groups were performed using the chi-square test. To account for potential sex-related confounding, additional median regression analyses at the 50th percentile were performed for each inflammatory index, with sex included as a covariate. Overall group effects were assessed using joint Wald tests, Bonferroni-adjusted pairwise comparisons were performed, and group-by-sex interactions were examined. Correlations between variables were assessed using Spearman correlation analysis. Univariable logistic regression was used to evaluate candidate variables associated with liver biochemical abnormalities, and variables with P < 0.10 were considered for multivariable analysis. Treatment status at the index assessment was retained in the multivariable model as a prespecified confounder. Sex was coded as male = 1 and female = 0, and treatment status as receiving ATD = 1 and not receiving ATD = 0. Because the inflammatory indices were measured on different scales, Z-score standardization was performed before logistic regression analysis to improve the comparability and interpretability of the effect estimates. The standardized variables were designated as ZMLR, ZSIRI, ZPIV, ZNLR, and ZSII. Because the inflammatory indices were mathematically related, they were evaluated separately and were not entered simultaneously into the same multivariable model. For the hyperthyroid relapse analysis, ROC curves were generated for TRAb and each inflammatory index. To avoid the simultaneous inclusion of mathematically related inflammatory indices, the inflammatory index with the highest individual AUC was selected and combined with TRAb to construct an exploratory two-predictor model. Optimal cutoff values were determined by maximizing the Youden index (sensitivity + specificity − 1). Multicollinearity was assessed using tolerance and the variance inflation factor (VIF), with tolerance ≤ 0.20 or VIF ≥ 5 considered indicative of potentially problematic collinearity. Model discrimination was evaluated using the area under the receiver operating characteristic curve (AUC), sensitivity, and specificity, whereas calibration was assessed using the Brier score, calibration slope, and calibration intercept. Internal validation was performed using 1,000 bootstrap resamples to estimate optimism-corrected performance, with repeated 10-fold cross-validation (100 repetitions) conducted as a sensitivity analysis. All statistical tests were two-sided, and *P* < 0.05 indicated statistical significance.

## 3 Results

### 3.1 Clinical baseline characteristics of 687 patients with GD

Baseline clinical and laboratory characteristics of the 687 patients with GD are summarized in **Table 1**. This study included 687 patients with GD, comprising 283 in the OH group, 137 in the SCH group, and 267 in the EU group. Sex distribution differed significantly among the three groups, with male patients accounting for 34.28%, 24.82%, and 21.35% of the OH, SCH, and EU groups, respectively (χ² = 12.110, *P* = 0.002). As expected, all thyroid-related parameters differed significantly across the three thyroid functional groups. Significant between-group differences were also observed in MONO, MPV, Hb, ALT, AST, TBIL, and DBIL, whereas age and the remaining hematological and liver biochemical parameters were comparable among the groups.

**Table 1:** Clinical and laboratory characteristics of patients with Graves’ disease stratified by thyroid functional status.

Variable	OH (n = 283)	SCH (n = 137)	EU (n = 267)	H/χ²/F	*P* value
Age (years)	37.00 (29.00, 49.00)	37.00 (29.00, 50.00)	38.00 (30.00, 50.00)	1.372	0.504
Male, n (%)	97 (34.28%)	34 (24.82%)	57 (21.35%)	12.110	**0.002**
TT3 (nmol/L)	2.98 (2.29, 4.22)	1.67 (1.44, 1.88)^**^	1.58 (1.39, 1.73)^**^	287.984	**<0.001**
TT4 (nmol/L)	184.10 (146.80, 244.25)	106.10 (85.65, 125.55)^**^	110.70 (89.20, 125.40)^**^	241.175	**<0.001**
TSH (μIU/mL)	0.007 (0.005, 0.011)	0.017 (0.009, 0.072)^**^	1.953 (1.071, 2.982)^**##^	488.849	**<0.001**
TRAb (IU/L)	10.70 (2.98, 24.20)	4.11 (2.04, 14.75)^**^	1.78 (1.19, 2.81)^**##^	204.012	**<0.001**
FT3 (pmol/L)	10.30 (7.43, 17.16)	5.44 (4.90, 5.87)^**^	5.07 (4.61, 5.52)^**##^	487.722	**<0.001**
FT4 (pmol/L)	29.19 (22.15, 46.88)	15.79 (13.39, 17.85)^**^	15.69 (13.97, 17.39)^**^	419.841	**<0.001**
WBC (×10^9^/L)	5.95 (4.91, 7.04)	5.76 (4.83, 6.94)	5.84 (4.88, 6.99)	0.037	0.982
PDW (%)	14.80 (10.90, 16.20)	15.70 (11.35, 16.20)	13.40 (11.00, 16.20)	2.119	0.347
MPV (fL)	10.00 (9.50, 10.90)	9.80 (9.30, 10.70)	10.20 (9.70, 10.90)^##^	6.968	**0.031**
PCT (%)	0.25 (0.23, 0.29)	0.24 (0.22, 0.29)	0.25 (0.21, 0.28)	5.339	0.069
P-LCR (%)	26.00 (22.00, 33.00)	24.00 (21.00, 31.00)	27.00 (22.00, 32.00)	4.581	0.101
NEUT (×10^9^/L)	3.19 (2.36, 4.11)	3.30 (2.61, 4.14)	3.32 (2.64, 4.09)	1.526	0.466
LYM (×10^9^/L)	2.00 (1.65, 2.44)	1.97 (1.63, 2.45)	2.03 (1.67, 2.42)	0.042	0.979
MONO (×10^9^/L)	0.43 (0.34, 0.54)	0.34 (0.28, 0.41)^**^	0.35 (0.29, 0.42)^**^	69.606	**<0.001**
Hb (g/L)	149.00 (138.00, 160.00)	145.00 (139.00, 156.50)	146.00 (135.00, 156.00)^*^	6.033	**0.049**
PLT (×10^9^/L)	253.94 ± 61.91	250.91 ± 70.85	243.43 ± 60.17	1.962	0.141
ALT (U/L)	22.00 (16.00, 32.00)	16.00 (12.00, 25.00)^**^	14.00 (9.50, 19.00)^**##^	72.121	**<0.001**
AST (U/L)	27.50 (22.00, 35.00)	27.00 (20.00, 33.00)	23.00 (19.00, 29.00)^**#^	25.302	**<0.001**
TBIL (μmol/L)	14.65 (11.10, 19.20)	14.10 (10.80, 18.70)	13.10 (10.35, 16.90)^**^	9.874	**0.007**
DBIL (μmol/L)	3.10 (2.13, 4.40)	3.00 (2.00, 4.30)	2.60 (1.70, 3.60)^**#^	16.232	**<0.001**
IBIL (μmol/L)	11.30 (8.43, 15.20)	11.10 (8.00, 14.60)	10.50 (7.85, 13.60)	5.710	0.058

Data are presented as mean ± SD, median (Q1, Q3), or n (%), as appropriate. Overall group comparisons were performed using one-way analysis of variance, the Kruskal–Wallis test, or Pearson’s chi-square test, as appropriate. For continuous variables with a statistically significant overall group difference, Bonferroni-adjusted pairwise comparisons were performed. All pairwise *P* values were Bonferroni-adjusted. *P < 0.05 and **P < 0.01 versus the OH group; #*P* < 0.05 and ##*P* < 0.01 versus the SCH group. Bold *P* values in the final column indicate statistically significant overall group differences at *P* < 0.05. SD, standard deviation; Q1, first quartile; Q3, third quartile; H, Kruskal–Wallis test statistic; χ², chi-square test statistic; F, one-way analysis-of-variance test statistic. OH, overt hyperthyroidism; SCH, subclinical hyperthyroidism; EU, euthyroid; TT3, total triiodothyronine; TT4, total thyroxine; TSH, thyroid-stimulating hormone; TRAb, thyrotropin receptor antibody; FT3, free triiodothyronine; FT4, free thyroxine; WBC, white blood cell count; PDW, platelet distribution width; MPV, mean platelet volume; PCT, plateletcrit; P-LCR, platelet large-cell ratio; NEUT, neutrophil count; LYM, lymphocyte count; MONO, monocyte count; Hb, hemoglobin; PLT, platelet count; ALT, alanine aminotransferase; AST, aspartate aminotransferase; TBIL, total bilirubin; DBIL, direct bilirubin; IBIL, indirect bilirubin.

The baseline characteristics of the OH-IP, OH-ATD, and OH-RP subgroups are presented in **Supplementary Table 2**. Significant differences were observed in thyroid function and selected hematological parameters, whereas age, sex, TRAb, and liver biochemical parameters were comparable among the three subgroups.

The baseline characteristics of the EU-ATD and EU-WD subgroups are presented in **Supplementary Table 3**. The two groups differed in TT4, TRAb, FT4, WBC, and NEUT, whereas no significant differences were observed in age, sex, or the remaining thyroid-related, hematological, and liver biochemical parameters.

### 3.2 Comparison of peripheral blood inflammatory indices across thyroid functional states and treatment stages in Graves’ disease

Because sex distribution differed among the groups, sex-adjusted median regression analyses were additionally performed. Significant overall group differences remained for MLR (adjusted Wald χ² = 30.390, *P* < 0.001), SIRI (adjusted Wald χ² = 16.603, *P* < 0.001), and PIV (adjusted Wald χ² = 13.418, *P* = 0.001), whereas no significant overall group differences were observed for NLR (adjusted Wald χ² = 3.533, *P* = 0.171) or SII (adjusted Wald χ² = 0.913, *P* = 0.634). Bonferroni-adjusted pairwise comparisons showed that MLR was higher in the OH group than in both the SCH and EU groups (both adjusted *P* < 0.001); SIRI was higher in the OH group than in the SCH (adjusted *P* < 0.001) and EU (adjusted *P* = 0.015) groups; and PIV was higher in the OH group than in the SCH (adjusted *P* = 0.007) and EU (adjusted *P* = 0.002) groups. No significant group-by-sex interactions were observed for MLR, SIRI, PIV, NLR, or SII (interaction *P* = 0.310, 0.876, 0.824, 0.793, and 0.840, respectively) (**Table 2**).

**Table 2:** Peripheral blood inflammatory indices across thyroid functional states: unadjusted and sex-adjusted comparisons.

Index	OH (n = 283)	SCH (n = 137)	EU (n = 267)	Unadjusted H	Unadjusted *P* value	Adjusted Wald χ²	Adjusted *P* value	Group × sex *P* value
MLR	0.22 (0.16, 0.28)	0.17 (0.14, 0.22)^**^	0.17 (0.14, 0.22)^**^	51.806	**<0.001**	30.390	**<0.001**	0.310
SIRI	0.69 (0.44, 0.99)	0.52 (0.39, 0.77)^**^	0.57 (0.41, 0.79)^*^	16.601	**<0.001**	16.603	**<0.001**	0.876
PIV	166.24 (102.48, 258.76)	132.57 (90.26, 187.44)^**^	130.58 (91.63, 209.78)^**^	18.811	**<0.001**	13.418	**0.001**	0.824
NLR	1.52 (1.16, 2.09)	1.65 (1.19, 2.11)	1.63 (1.26, 2.09)	2.741	0.254	3.533	0.171	0.793
SII	392.09 (268.62, 539.15)	406.42 (278.82, 528.39)	371.63 (277.97, 528.25)	0.164	0.921	0.913	0.634	0.840

Unadjusted comparisons used the Kruskal-Wallis test. Sex-adjusted median regression was additionally performed. *P < 0.05 and **P < 0.01 versus the OH group. Bold *P* values indicate statistical significance at *P* < 0.05.

After stratification of patients with OH according to treatment stage into the OH-IP, OH-ATD, and OH-RP groups, significant differences were observed in MLR, SIRI, PIV, NLR, and SII among the three groups (all *P* ≤ 0.001). All five inflammatory indices were increased in the OH-RP group relative to the OH-IP and OH-ATD groups in pairwise comparisons (all *P* < 0.01). In addition, NLR was significantly higher in the OH-ATD group than in the OH-IP group (*P* < 0.05), whereas the remaining indices were comparable between the OH-IP and OH-ATD groups (all *P* > 0.05) (**Table 3A**).

**Table 3:** Peripheral blood inflammatory indices stratified by treatment status within the OH and EU groups.

A. OH group					
Index	OH-IP (n = 54)	OH-ATD (n = 191)	OH-RP (n = 38)	H	*P* value
MLR	0.22 (0.16, 0.28)	0.20 (0.16, 0.26)	0.27 (0.25, 0.35)^**##^	30.872	**<0.001**
SIRI	0.67 (0.43, 0.90)	0.62 (0.41, 0.90)	1.07 (0.87, 1.22)^**##^	32.335	**<0.001**
PIV	170.43 (100.91, 246.11)	153.98 (98.92, 241.77)	270.45 (178.71, 366.18)^**##^	24.660	**<0.001**
NLR	1.30 (1.01, 1.77)	1.53 (1.19, 2.02)^*^	2.20 (1.50, 2.63)^**##^	22.493	**<0.001**
SII	323.68 (234.27, 503.88)	382.64 (264.90, 535.42)	521.68 (386.04, 673.07)^**##^	14.977	**0.001**
B. EU group					
Index	EU-ATD (n = 170)		EU-WD (n = 97)	H	*P* value
MLR	0.18 (0.14, 0.23)		0.16 (0.14, 0.21)	1.467	0.226
SIRI	0.62 (0.42, 0.90)		0.53 (0.39, 0.69)	7.232	**0.007**
PIV	142.76 (92.28, 240.33)		119.28 (89.82, 160.22)	7.728	**0.005**
NLR	1.70 (1.29, 2.17)		1.51 (1.19, 1.92)	8.550	**0.003**
SII	408.66 (304.73, 578.98)		331.32 (272.99, 459.09)	9.638	**0.002**

Data are presented as median (IQR). For panel A, *P < 0.05 and **P < 0.01 versus the OH-IP group; #*P* < 0.05 and ##*P* < 0.01 for the comparison between the OH-ATD and OH-RP groups. Bold P values indicate statistical significance at P < 0.05.

Among patients in the EU subgroup, the EU-ATD group showed higher SIRI, PIV, NLR, and SII levels than the EU-WD group (*P* = 0.007, *P* = 0.005, *P* = 0.003, and *P* = 0.002, respectively), whereas no significant difference in MLR was observed between the two groups (*P* = 0.226) (**Table 3B**).

### 3.3 Correlations of inflammatory indices with clinical characteristics and biochemical variables in patients with OH

Spearman correlation analysis was performed to evaluate the associations of inflammatory indices with baseline clinical characteristics, thyroid function, and liver biochemical parameters in patients with OH. For baseline clinical characteristics and thyroid function, positive correlations were observed between MLR and age (r_s_ = 0.152, *P* = 0.017), FT3 (r_s_ = 0.206, *P* = 0.001), and FT4 (r_s_ = 0.181, P = 0.004). NLR was positively correlated with TSH (r_s_ = 0.193, *P* = 0.002) but negatively correlated with FT3 (rs = -0.166, *P* = 0.009) and FT4 (r_s_ = -0.174, *P* = 0.006). In addition, SII was positively correlated with TSH (rs = 0.135, *P* = 0.034) and negatively correlated with FT3 (r_s_ = -0.131, *P* = 0.039). No significant correlations were observed between SIRI or PIV and any baseline clinical or thyroid function parameter (all *P* > 0.05; **Table 4A**).

**Table 4:** Spearman correlations of inflammatory indices with clinical and biochemical variables in the OH group.

A. Clinical characteristics and thyroid function parameters										
Index	Age		TSH		TRAb		FT3		FT4	
	r_s_	*P* value	r_s_	*P* value	r_s_	*P* value	r_s_	*P* value	r_s_	*P* value
MLR	**0.152**	**0.017**	0.004	0.955	0.037	0.566	**0.206**	**0.001**	**0.181**	**0.004**
SIRI	0.116	0.069	0.106	0.096	−0.057	0.372	0.017	0.790	−0.011	0.859
PIV	−0.003	0.956	0.083	0.195	−0.027	0.671	0.015	0.817	0.006	0.920
NLR	0.116	0.068	**0.193**	**0.002**	−0.060	0.349	**−0.166**	**0.009**	**−0.174**	**0.006**
SII	−0.017	0.794	**0.135**	**0.034**	−0.026	0.679	**−0.131**	**0.039**	−0.124	0.051
B. Liver biochemical parameters										
Index	ALT		AST		TBIL		DBIL		IBIL	
	r_s_	*P* value	r_s_	*P* value	r_s_	*P* value	r_s_	*P* value	r_s_	*P* value
MLR	**0.206**	**0.001**	**0.194**	**0.002**	0.043	0.496	0.057	0.371	0.037	0.558
SIRI	0.095	0.137	0.027	0.668	−0.037	0.561	−0.020	0.752	−0.042	0.512
PIV	0.067	0.292	0.016	0.803	−0.072	0.262	−0.035	0.580	−0.076	0.234
NLR	−0.071	0.263	−0.082	0.197	−0.005	0.939	−0.037	0.558	−0.009	0.889
SII	−0.057	0.369	−0.076	0.232	−0.065	0.304	−0.079	0.213	−0.061	0.335

Spearman rank correlation analysis was performed for continuous variables. Values are presented as Spearman’s correlation coefficients (r_s_) and corresponding two-sided *P* values. Bold correlation coefficients and corresponding *P* values indicate statistically significant correlations at *P* < 0.05.

With respect to liver biochemical parameters, MLR was positively correlated with ALT (r_s_ = 0.206, *P* = 0.001) and AST (r_s_ = 0.194, *P* = 0.002) but was not significantly correlated with TBIL, DBIL, or IBIL (all *P* > 0.05). None of the five liver biochemical parameters were significantly correlated with SIRI, PIV, NLR, or SII (all *P* > 0.05; **Table 4B**).

### 3.4 Risk factors and model discrimination for liver biochemical abnormalities in patients with GD during the overt hyperthyroid stage

In the univariable logistic regression analysis, sex (OR = 0.416, 95% CI: 0.241–0.719, *P* = 0.002), TRAb (OR = 1.033, 95% CI: 1.009–1.057, *P* = 0.006), Hb (OR = 1.030, 95% CI: 1.015–1.046, *P* < 0.001), and ZMLR (OR = 1.361, 95% CI: 1.045–1.773, *P* = 0.022) were significantly associated with liver biochemical abnormalities. FT4 met the prespecified criterion for entry into the multivariable model (*P* = 0.076) and was therefore also included. Treatment status was not significant (OR = 1.249, 95% CI: 0.733–2.129, *P* = 0.413) but was retained as a prespecified confounder (**Table 5**).

**Table 5:** Univariable and multivariable logistic regression analyses of factors associated with liver biochemical abnormalities in the OH cohort.

Variable	Univariable		Multivariable	
	OR (95% CI)	*P* value	OR (95% CI)	*P* value
Sex	**0.416 (0.241–0.719)**	**0.002**	0.900 (0.421–1.925)	0.786
Age	1.014 (0.993–1.034)	0.189		
Treatment status	1.249 (0.733–2.129)	0.413	0.882 (0.473–1.643)	0.692
TSH	0.925 (0.754–1.136)	0.457		
TRAb	**1.033 (1.009–1.057)**	**0.006**	**1.032 (1.007–1.059)**	**0.014**
FT3	1.019 (0.989–1.049)	0.211		
FT4	1.007 (0.999–1.015)	0.076	1.008 (0.998–1.017)	0.112
WBC	0.982 (0.845–1.142)	0.817		
PDW	0.995 (0.911–1.086)	0.907		
MPV	1.097 (0.885–1.359)	0.400		
PCT	1.146 (0.716–1.834)	0.570		
P-LCR	1.007 (0.978–1.036)	0.652		
NEUT	0.985 (0.808–1.200)	0.879		
LYM	0.875 (0.611–1.252)	0.465		
MONO	3.477 (0.629–19.222)	0.153		
Hb	**1.030 (1.015–1.046)**	**<0.001**	**1.034 (1.013–1.056)**	**0.001**
PLT	0.997 (0.993–1.001)	0.105		
ZMLR	**1.361 (1.045–1.773)**	**0.022**	**1.377 (1.027–1.846)**	**0.032**
ZSIRI	1.235 (0.949–1.607)	0.116		
ZPIV	1.111 (0.856–1.442)	0.430		
ZNLR	1.136 (0.881–1.466)	0.326		
ZSII	1.021 (0.795–1.311)	0.872		

Variables with *P* < 0.10 in the univariable analysis were considered for inclusion in the multivariable model. Treatment status was retained as a prespecified potential confounder. Sex was coded as male = 1 and female = 0; treatment status was coded as receiving ATD therapy = 1 and not receiving ATD therapy = 0. Bold estimates and *P* values indicate statistical significance at *P* < 0.05.

Collinearity diagnostics indicated no problematic multicollinearity among the variables included in the full multivariable model (tolerance, 0.547–0.938; VIF, 1.066–1.827; **Supplementary Table 4**). In the multivariable logistic regression model including sex, FT4, treatment status, TRAb, Hb, and ZMLR, TRAb (adjusted OR = 1.032, 95% CI: 1.007–1.059, *P* = 0.014), Hb (adjusted OR = 1.034, 95% CI: 1.013–1.056, *P* = 0.001), and ZMLR (adjusted OR = 1.377, 95% CI: 1.027–1.846, *P* = 0.032) remained independently associated with liver biochemical abnormalities, whereas sex, FT4, and treatment status were not statistically significant (all *P* > 0.05) (**Table 5**). TRAb, Hb, and ZMLR were subsequently incorporated into the final prediction model. No problematic multicollinearity was identified among these three predictors in the final TRAb–Hb–ZMLR prediction model (tolerance, 0.995–0.997; VIF, 1.003–1.005; **Supplementary Table 4**).

**Figure 2:**
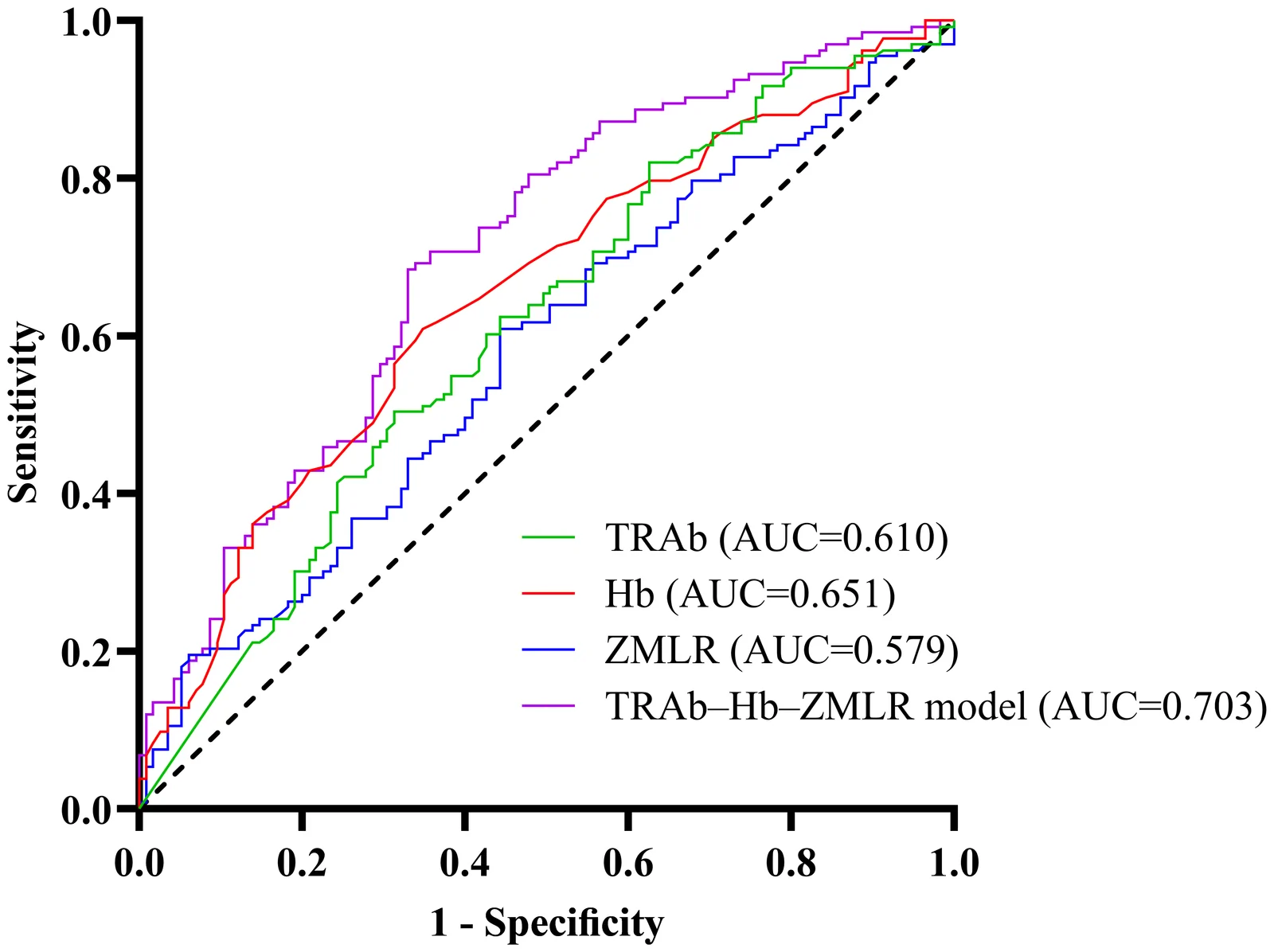
Receiver operating characteristic curves of TRAb, Hb, ZMLR, and the combined TRAb–Hb–ZMLR model for discriminating liver biochemical abnormalities in patients with overt hyperthyroidism with complete liver biochemical data (n = 248).

**Figure 3:**
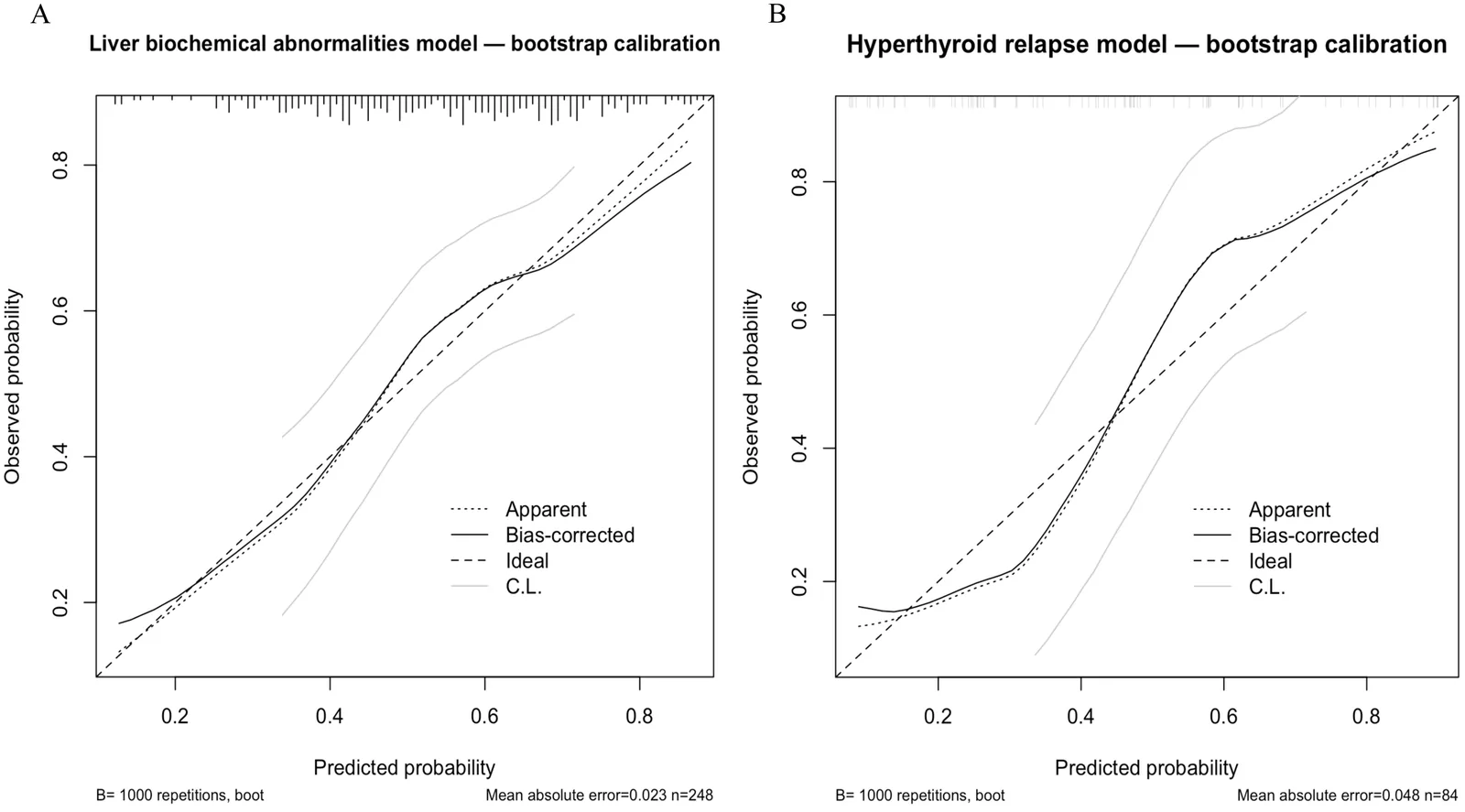
Bootstrap calibration curves for the liver biochemical abnormalities and hyperthyroid relapse models. **(A)** Calibration of the TRAb–Hb–ZMLR model for liver biochemical abnormalities in the OH subgroup with complete liver biochemical data (n = 248). **(B)** TRAb–PIV model for 12-month hyperthyroid relapse in the EU-WD cohort with complete follow-up data (n = 84). Dotted lines indicate apparent calibration, solid lines indicate bootstrap bias-corrected calibration based on 1,000 resamples, and dashed diagonal lines indicate ideal calibration. Light-gray lines represent confidence limits, and rug marks show the distribution of predicted probabilities.

The combined TRAb–Hb–ZMLR model had an apparent AUC of 0.703 (95% CI: 0.639–0.768) (**Table 6A**; **Figure 2**). Bootstrap validation yielded an optimism-corrected AUC of 0.692, a corrected Brier score of 0.224, a calibration slope of 0.949, and a calibration intercept of 0.004. Repeated 10-fold cross-validation yielded an AUC of 0.680 and a calibration slope of 0.843 (**Table 6C**; **Figure 3A**). Overall, the model demonstrated limited-to-moderate internally validated discrimination; however, the relatively small cohort and lack of external validation preclude conclusions regarding its clinical utility.

**Table 6:** Discrimination and internal validation of models for liver biochemical abnormalities and hyperthyroid relapse.

A. Liver biochemical abnormalities model in the OH cohort (n = 248)								
Predictor/model	Cutoff	Youden index	AUC	95% CI	Sensitivity	Specificity	*P* value	
TRAb	3.125	0.194	**0.610**	0.539–0.680	0.820	0.374	**0.003**	
Hb	149.5	0.261	**0.651**	0.583–0.719	0.609	0.652	**<0.001**	
ZMLR	−0.226	0.166	**0.579**	0.508–0.650	0.609	0.557	**0.032**	
TRAb–Hb–ZMLR model	0.537	0.354	**0.703**	0.639–0.768	0.684	0.670	**<0.001**	
B. Hyperthyroid relapse model in the EU-WD subgroup (n = 84)								
TRAb	1.515	0.371	**0.701**	0.587–0.815	0.632	0.739		**0.002**
MLR	0.139	0.259	0.604	0.483–0.726	0.868	0.391		0.102
NLR	1.273	0.255	0.609	0.488–0.731	0.842	0.413		0.086
SIRI	0.435	0.294	**0.644**	0.526–0.762	0.816	0.478		**0.024**
SII	374.912	0.362	**0.708**	0.596–0.819	0.579	0.783		**0.001**
PIV	129.994	0.445	**0.734**	0.627–0.841	0.684	0.761		**<0.001**
TRAb–PIV model	0.538	0.549	**0.815**	0.716–0.913	0.658	0.891		**<0.001**
C. Internal validation of the two combined models								
Model	Metric	Apparent performance		Bootstrap optimism	Bootstrap-corrected performance			Repeated 10-fold CV performance
Liver biochemical abnormalities model	AUC	0.703		0.012	0.692			0.680
Liver biochemical abnormalities model	Calibration slope	1.000		0.051	0.949			0.843
Liver biochemical abnormalities model	Calibration intercept	0.000		−0.004	0.004			—
Liver biochemical abnormalities model	Brier score	0.217		−0.007	0.224			—
Hyperthyroid relapse model	AUC	0.815		0.010	0.805			0.788
Hyperthyroid relapse model	Calibration slope	1.000		0.045	0.955			0.758
Hyperthyroid relapse model	Calibration intercept	0.000		0.008	−0.008			—
Hyperthyroid relapse model	Brier score	0.175		−0.013	0.188			—

Cutoff values for individual predictors were determined by maximizing the Youden index, whereas the cutoff for each combined model represents the predicted-probability threshold. The P values in sections A and B were derived from tests of whether the AUC differed from 0.5. Bootstrap internal validation was performed using 1,000 nonparametric resamples. Bootstrap-corrected performance was calculated as the apparent performance minus the estimated bootstrap optimism. Repeated 10-fold cross-validation was performed with 100 repetitions using out-of-fold predictions. Bold values indicate statistical significance at *P* < 0.05. A dash indicates that the corresponding metric was not estimated.

### 3.5 Discriminatory performance of inflammatory markers for hyperthyroid relapse in the EU-WD subgroup with complete follow-up

ROC curve analysis was performed to assess the discrimination of individual markers for hyperthyroid relapse among 84 patients in the EU-WD cohort with complete 12-month follow-up data. TRAb, SIRI, SII, and PIV all showed statistically significant discrimination (all P < 0.05), whereas MLR and NLR did not (both *P* > 0.05) (**Table 6B**).

**Figure 4:**
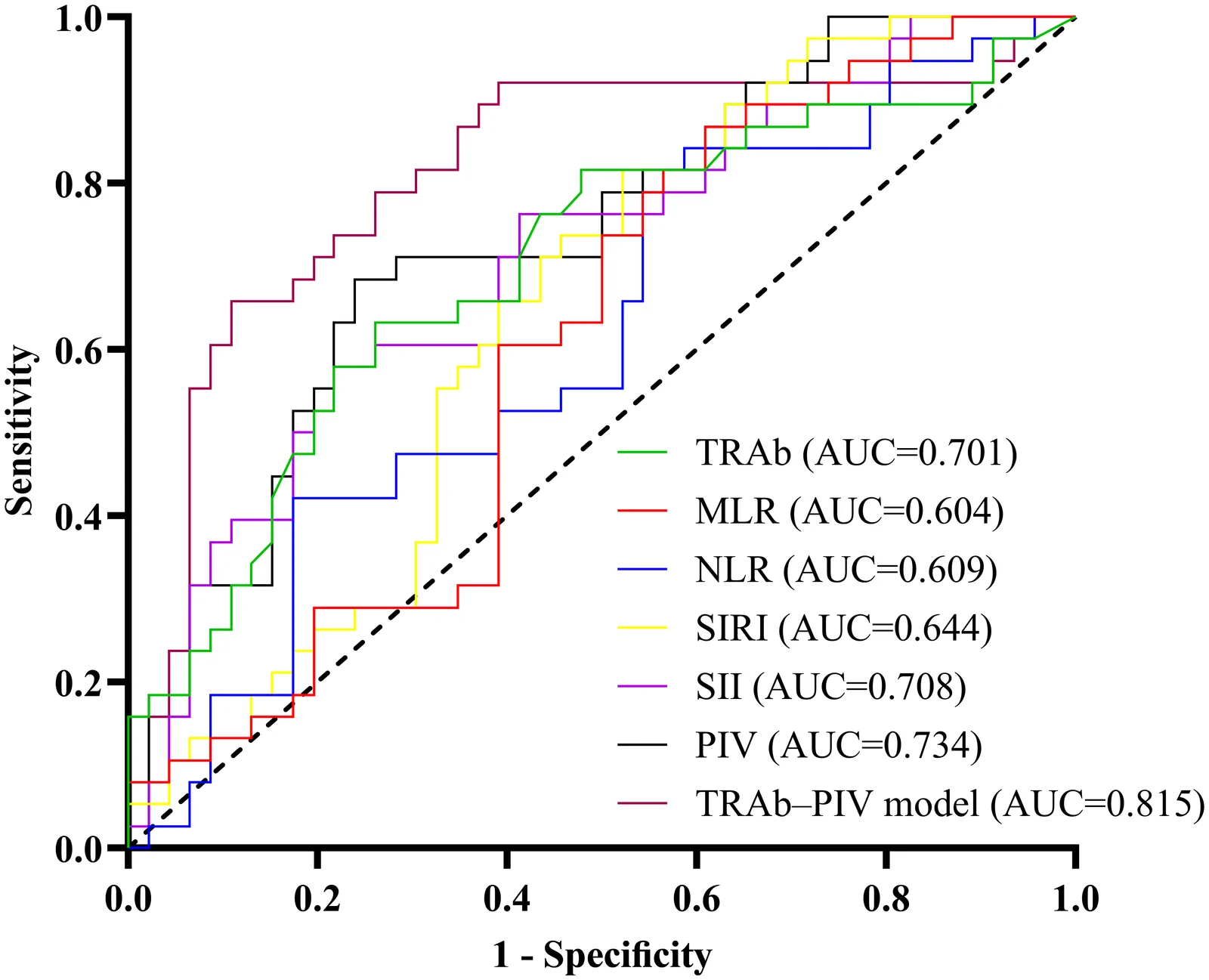
Receiver operating characteristic curves of TRAb, the five peripheral blood inflammatory indices, and the combined TRAb–PIV model for discriminating hyperthyroid relapse during the 12-month follow-up in the EU-WD cohort (n = 84).

Among the individual markers, PIV showed the highest discrimination, with an area under the curve (AUC) of 0.734 (95% CI: 0.627–0.841), an optimal cutoff value of 129.994, a sensitivity of 0.684, and a specificity of 0.761. TRAb, SIRI, and SII also showed significant discrimination, with AUCs of 0.701, 0.644, and 0.708, respectively. The combined TRAb–PIV model showed better apparent discrimination, with an AUC of 0.815 (95% CI: 0.716–0.913), a sensitivity of 0.658, a specificity of 0.891, and a Youden index of 0.549 (**Table 6B**; **Figure 4**).

Bootstrap validation yielded an optimism-corrected AUC of 0.805, a corrected Brier score of 0.188, a calibration slope of 0.955, and a calibration intercept of −0.008. Repeated 10-fold cross-validation yielded an AUC of 0.788 and a calibration slope of 0.758 (**Table 6C**; **Figure 3B**). No problematic collinearity was observed between TRAb and PIV (tolerance = 0.997; VIF = 1.003; **Supplementary Table 4**). Although the model retained relatively good discrimination after internal validation, its clinical utility requires external validation in larger cohorts.

## 4 Discussion

The present study yielded three main findings. First, after adjustment for sex, MLR, SIRI, and PIV were higher in patients with overt hyperthyroidism than in those with subclinical hyperthyroidism or euthyroidism, whereas NLR and SII did not differ significantly across thyroid functional states. Second, MLR was positively correlated with ALT and AST, while TRAb, hemoglobin, and ZMLR were independently associated with liver biochemical abnormalities; the multivariable model incorporating these factors showed limited-to-moderate discrimination after internal validation. Third, PIV showed the highest individual discrimination for hyperthyroid relapse, and the combined TRAb–PIV model retained relatively good discrimination after internal validation.

The pattern of NLR alteration in GD appears to differ from the elevated NLR commonly observed in many systemic autoimmune diseases. NLR has been reported to be elevated in GO and to correlate with clinical activity score and disease activity (22, 23), whereas some studies in isolated GD have reported lower NLR levels than in healthy controls and negative correlations with FT3 and FT4 (20, 21). Our findings are consistent with the latter observations and suggest that NLR may be influenced by thyrotoxicosis-related shifts in leukocyte composition, which may limit its value as a stable inflammatory marker in isolated GD. The contrasting findings between GO and isolated GD may partly reflect differences between predominantly local orbital inflammation and the combined autoimmune and thyrotoxic influences in GD. In contrast, MLR, SIRI, and PIV were higher in the OH group than in the SCH and EU groups, and these differences remained significant after adjustment for sex. Together with prior evidence that these markers are elevated in GD compared with healthy controls (19), our findings suggest that monocyte-containing indices show clearer differences across thyroid functional states and may provide complementary information on inflammation-related changes in GD. A plausible explanation for these findings is the involvement of monocytes in GD immunopathology. Peripheral CD14++CD16+ proinflammatory monocytes are expanded and activated in GD and may induce Th1 inflammatory responses by secreting IL-12 (32, 33). Furthermore, monocytes may also act as TSH receptor effector cells and, upon TRAb stimulation, produce IL-6, IL-8, and reactive oxygen species (ROS), thereby further amplifying the inflammatory cascade (34). In addition, monocyte infiltration is increased in thyroid tissue from GD patients and may migrate to local inflammatory sites through a GPR15-related homing pathway (32, 35). Evidence from GO further suggests that recruited monocytes can differentiate into proinflammatory macrophages and contribute to tissue remodeling (36, 37). Moreover, monocytes may enhance B-cell activation through increased expression of B-cell activating factor (BAFF) or differentiate into dendritic cells that further promote T follicular helper cell-mediated B-cell activation and autoantibody production (32, 38). Collectively, these mechanisms provide a biological basis for the clearer differences observed in MLR, SIRI, and PIV across thyroid functional states.

The association between MLR and liver biochemical abnormalities further suggests that monocyte-related inflammation may be relevant to extrathyroidal involvement in GD. Our results showed that, in GD patients during the hyperthyroid phase, MLR was positively correlated with ALT and AST levels, while TRAb, hemoglobin, and ZMLR remained independently associated with liver biochemical abnormalities. Similar associations between MLR and liver enzyme elevation, hepatic steatosis, or fibrosis risk have been reported in psoriasis and metabolic dysfunction-associated steatotic liver disease (39, 40). Liver biochemical abnormalities are common in patients with GD, and their pathogenesis is complex. In addition to direct hepatocellular injury caused by thyrotoxicosis and congestive hepatic damage secondary to hyperthyroidism-related cardiac dysfunction, relative ischemia/hypoxia under a hypermetabolic state, enhanced oxidative stress, and immune-inflammatory responses may all contribute (41). Ischemic-hypoxic injury may induce the liver to release chemotactic signals such as CCL2, thereby recruiting inflammatory monocytes and promoting their differentiation into proinflammatory macrophages, which in turn aggravate liver injury through ROS and multiple proinflammatory and profibrotic mediators (42, 43). On the other hand, monocytes may also participate in immune dysregulation by promoting BAFF expression, enhancing B-cell activation, and facilitating TRAb production (32). Autoimmune mechanisms have been proposed to contribute to GD-related hepatic injury, and the expression of TSH receptors on hepatocytes provides a possible biological basis for direct hepatic effects of TRAb, although this mechanism remains unproven (44, 45). Therefore, elevated MLR may reflect a monocyte-associated immune-inflammatory process involved in GD-related liver biochemical abnormalities rather than serving solely as a nonspecific marker of systemic inflammation.

Persistent immune activation may also contribute to hyperthyroid relapse after ATD withdrawal. Although antithyroid drugs can effectively control thyrotoxicosis and restore thyroid function, they often fail to completely correct the underlying immune abnormality. Therefore, if TRAb remains positive or rises again after drug withdrawal, the TSH receptor signaling pathway may be reactivated, leading to relapse (2). Previous studies have linked hyperthyroid relapse to persistent immune activation. Elevated serum IL-17 at ATD withdrawal was identified as an independent risk factor for relapse (46), while activated Th17 cell levels increased in parallel with the Graves’ Recurrent Events After Therapy (GREAT) score and remained elevated after ATD treatment (47). In addition, SIGLEC1 positivity at ATD withdrawal was associated with a higher cumulative risk of relapse (48). In the present cohort of patients who were off ATD therapy and biochemically euthyroid at the index assessment, PIV showed the highest individual discrimination for subsequent relapse. As a composite inflammatory index integrating neutrophils, platelets, monocytes, and lymphocytes, PIV may reflect the overall status of innate immune activation, platelet-mediated inflammatory amplification, and adaptive immune dysregulation more comprehensively than single or relatively simplified indices such as NLR and SII. Its prognostic or discriminatory value has also been reported in other inflammatory clinical settings (49, 50), although evidence in GD remains limited and has primarily focused on NLR (51). Combining PIV with TRAb improved discrimination in our cohort, suggesting potentially complementary information: TRAb reflects GD-specific humoral autoimmunity, whereas PIV captures broader systemic inflammatory activity and immune-cell composition. Nevertheless, the model remains exploratory and requires external validation before clinical application.

However, this study has some limitations. First, its single-center retrospective design may have introduced selection bias and precluded causal inference. Disease duration, cumulative ATD exposure, actual ATD dose, and medication adherence were incompletely documented, leaving residual confounding despite adjustment for treatment status. Second, because this study was based on real-world outpatient medical records, laboratory testing was determined by clinical indications and was not fully standardized. Albumin, globulins, alkaline phosphatase, gamma-glutamyl transferase, C-reactive protein, erythrocyte sedimentation rate, cytokine profiles, and other direct inflammatory markers were unavailable for a substantial proportion of patients. Although thyroid functional classification was based on FT3, FT4, and TSH rather than total thyroid hormone concentrations, the absence of thyroid hormone-binding proteins and direct inflammatory markers limited further interpretation of the observed associations. Future prospective studies should incorporate standardized and more comprehensive laboratory assessments. Third, peripheral blood inflammatory indices were not measured at a standardized visit coinciding with ATD withdrawal, and the interval between ATD withdrawal and the index assessment could not be consistently determined. Therefore, the hyperthyroid relapse model estimates discrimination for events occurring during the 12 months after the index assessment among patients who were off ATDs and biochemically euthyroid at that assessment, rather than cumulative relapse risk from the date of ATD withdrawal. Fourth, both models were developed and internally validated in the same cohorts. Bootstrap and repeated cross-validation reduced but did not eliminate concerns about overfitting, particularly because the hyperthyroid relapse cohort was small and predictor selection was data-driven. Statistically significant ROC results and internal validation do not establish clinical utility. The models and cutoff values should therefore be considered exploratory and require prospective multicenter external validation before routine clinical use.

In conclusion, monocyte-containing peripheral blood inflammatory indices may provide readily available adjunctive information for inflammatory assessment and exploratory risk stratification in GD, although their clinical utility requires prospective external validation.

## Data Availability

The original contributions presented in the study are included in the article/**Supplementary Material**. Further inquiries can be directed to the corresponding author.

## References

[1] Smith TJ Hegedüs L . Graves’ disease. N Engl J Med. (2016) 375:1552–65. doi: 10.1056/NEJMra1510030 27797318

[2] Lanzolla G Marinò M Menconi F . Graves disease: latest understanding of pathogenesis and treatment options. Nat Rev Endocrinol. (2024) 20:647–60. doi: 10.1038/s41574-024-01016-5 39039206

[3] Tomczyńska M Saluk-Bijak J . The mutual cooperation of blood platelets and lymphocytes in the development of autoimmune thyroid diseases. Acta Biochim Pol. (2018) 65:17–24. doi: 10.18388/abp.2017_2321 29549672

[4] Fallahi P Ferrari SM Elia G Ragusa F Paparo SR Patrizio A . Cytokines as targets of novel therapies for Graves' ophthalmopathy. Front Endocrinol (Lausanne). (2021) 12:654473. doi: 10.3389/fendo.2021.654473 33935970 PMC8085526

[5] Rapoport B McLachlan SM . Graves' hyperthyroidism is antibody-mediated but is predominantly a Th1-type cytokine disease. J Clin Endocrinol Metab. (2014) 99:4060–1. doi: 10.1210/jc.2014-3011 25210884 PMC4223433

[6] Ueland HO Ulvik A Løvås K Wolff ASB Breivik LE Stokland AEM . Systemic activation of the kynurenine pathway in Graves disease with and without ophthalmopathy. J Clin Endocrinol Metab. (2023) 108:1290–7. doi: 10.1210/clinem/dgad004 36611247 PMC10188306

[7] Popławska-Kita A Siewko K Telejko B Modzelewska A Myśliwiec J Milewski R . The changes in the endothelial function and haemostatic and inflammatory parameters in subclinical and overt hyperthyroidism. Int J Endocrinol. (2013) 2013:981638. doi: 10.1155/2013/981638 24367378 PMC3866785

[8] Neves C Neves JS Pereira LM Sokhatska O Queirós J Delgado JL . Interrelationships between thyroid function, autoimmunity, low-grade inflammation, lipid profile and insulin resistance in Graves' disease. Endocr Abstr. (2024) 99:P380. doi: 10.1530/endoabs.99.P380 11032665

[9] Yoldas A Bahtiyar N Aydemir B Toplan S . Evaluation of selenoproteins and proinflammatory cytokines in L-thyroxine-induced hyperthyroid rats: effects of selenium supplementation. Arch Endocrinol Metab. (2025) 69:e240444. doi: 10.20945/2359-4292-2024-0444 40271987 PMC12017630

[10] Al-Kuraishy HM Sulaiman GM Mohammed HA Abu-Alghayth MH Albukhaty S Jabir MS . The role of autophagy in Graves disease: knowns and unknowns. Front Cell Dev Biol. (2025) 12:1480950. doi: 10.3389/fcell.2024.1480950 39834383 PMC11743935

[11] Yasin A İlhan MM Turan S Yaylım İ Karaman Ö Taşan E . The relationship of Graves disease with TNF-α, galectin-3 and fibronectin levels. J Tepecik Educ Res Hosp. (2020) 30:246–51. doi: 10.5222/terh.2020.86719

[12] Huang Y Liu A Liang L Jiang J Luo H Deng W . Diagnostic value of blood parameters for community-acquired pneumonia. Int Immunopharmacol. (2018) 64:10–5. doi: 10.1016/j.intimp.2018.08.022 30144639

[13] Bilen MA Martini DJ Liu Y Lewis C Collins HH Shabto JM . The prognostic and predictive impact of inflammatory biomarkers in patients who have advanced-stage cancer treated with immunotherapy. Cancer. (2019) 125:127–34. doi: 10.1002/cncr.31778 30329148

[14] Hu B Yang XR Xu Y Sun YF Sun C Guo W . Systemic immune-inflammation index predicts prognosis of patients after curative resection for hepatocellular carcinoma. Clin Cancer Res. (2014) 20:6212–22. doi: 10.1158/1078-0432.CCR-14-0442 25271081

[15] Qi Q Zhuang L Shen Y Geng Y Yu S Chen H . A novel systemic inflammation response index (SIRI) for predicting the survival of patients with pancreatic cancer after chemotherapy. Cancer. (2016) 122:2158–67. doi: 10.1002/cncr.30057 27152949

[16] Fucà G Guarini V Antoniotti C Morano F Moretto R Corallo S . The pan-immune-inflammation value is a new prognostic biomarker in metastatic colorectal cancer: results from a pooled-analysis of the Valentino and TRIBE first-line trials. Br J Cancer. (2020) 123:403–9. doi: 10.1038/s41416-020-0894-7 32424148 PMC7403416

[17] Li D Wu M . Pattern recognition receptors in health and diseases. Signal Transduct Target Ther. (2021) 6:291. doi: 10.1038/s41392-021-00687-0 34344870 PMC8333067

[18] Koupenova M Livada AC Morrell CN . Platelet and megakaryocyte roles in innate and adaptive immunity. Circ Res. (2022) 130:288–308. doi: 10.1161/CIRCRESAHA.121.319821 35050690 PMC8852355

[19] Soyer AK Cuhaci Seyrek FN Demirel KD Tam AA Topaloglu O Ersoy R . The role of blood cell-derived parameters in the differential diagnosis of subacute thyroiditis and Graves’ disease and long-term outcomes in subacute thyroiditis. Endocr Res. (2025) 50:163–74. doi: 10.1080/07435800.2025.2505627 40372793

[20] Dağdeviren M Akkan T Yapar D Karakaya S Dağdeviren T Ertuğrul D . Can neutrophil/lymphocyte ratio be used as an indicator of inflammation in patients with hyperthyroidism? J Med Biochem. (2020) 39:7–12. doi: 10.2478/jomb-2019-0004 32549771 PMC7282227

[21] Turan E . Evaluation of neutrophil-to-lymphocyte ratio and hematologic parameters in patients with Graves' disease. Bratisl Lek Listy. (2019) 120:476–80. doi: 10.4149/BLL_2019_076 31223030

[22] Niu T Wang L Deng J Shi Y Liu Y Tong B . Combining biomarkers to predict the disease activity of Graves’ ophthalmopathy: a combinatory model of the NLR, TRAb and FT4. Front Endocrinol (Lausanne). (2025) 16:1546211. doi: 10.3389/fendo.2025.1546211 40620798 PMC12226272

[23] Yilmaz Tugan B Ergen A Ozkan B . Monocyte-to-high-density lipoprotein ratio and systemic immune-inflammation index: potential parameters for the evaluation of disease activity and severity in Graves' ophthalmopathy? Int Ophthalmol. (2024) 44:154. doi: 10.1007/s10792-024-03077-x 38509387

[24] Yuksel N Saritas O Yuksel E . Effect of thyroid hormone status on complete blood cell count-derived inflammatory biomarkers in patients with moderate-to-severe Graves' ophthalmopathy. Int Ophthalmol. (2023) 43:3355–62. doi: 10.1007/s10792-023-02742-x 37209204

[25] Yoon D Yun C Beerman I Beydoun MA Launer LJ Song M . Elevated neutrophil-to-lymphocyte ratio and the incidence of autoimmune diseases: evidence from a large prospective cohort study. Sci Rep. (2026) 16:667. doi: 10.1038/s41598-025-21188-y 41495095 PMC12780031

[26] Yu R Zhang L Zhang J Long T Li J Zou Y . The diagnostic and prognostic role of novel biomarkers in anti-neutrophil cytoplasmic antibody-associated vasculitis. Front Immunol. (2025) 16:1588287. doi: 10.3389/fimmu.2025.1588287 40599788 PMC12209192

[27] Zhang J Wang Y Nie W Li Q Li SG Jin D . CBC-derived inflammatory indices for rheumatoid arthritis diagnosis and activity assessment: differential performance by serostatus. Front Immunol. (2026) 17:1740898. doi: 10.3389/fimmu.2026.1740898 41646848 PMC12867769

[28] Scappaticcio L Longo M Maiorino MI Pernice V Caruso P Esposito K . Abnormal liver blood tests in patients with hyperthyroidism: systematic review and meta-analysis. Thyroid. (2021) 31:884–94. doi: 10.1089/thy.2020.0715 33327837

[29] Kahaly GJ Bartalena L Hegedüs L Leenhardt L Poppe K Pearce SH . 2018 European Thyroid Association guideline for the management of Graves' hyperthyroidism. Eur Thyroid J. (2018) 7:167–86. doi: 10.1159/000490384 30283735 PMC6140607

[30] Takizawa H Uchida T Suzuki N Onose H Yamada E Hashimoto K . Influence of therapeutic inorganic iodine on long-term prognosis of Graves' disease: a multicenter prospective observational study. Endocr J. (2025) 72:663–70. doi: 10.1507/endocrj.EJ24-0575 39993757 PMC12171157

[31] Thewjitcharoen Y Karndumri K Chatchomchuan W Porramatikul S Krittiyawong S Wanothayaroj E . Serum T3 level and duration of minimum maintenance dose therapy predict relapse in methimazole-treated Graves disease. J Endocr Soc. (2021) 5:bvaa170. doi: 10.1210/jendso/bvaa170 33305160 PMC7716657

[32] Chen X Wang Y Qi Y Yan J Huang F Zhou M . Expansion of inflammatory monocytes in periphery and infiltrated into thyroid tissue in Graves' disease. Sci Rep. (2021) 11:13443. doi: 10.1038/s41598-021-92737-4 34188092 PMC8242071

[33] Yin Q Song D Chen J Ning G Wang W Wang S . The CD14++CD16+ monocyte subset is expanded and controls Th1 cell development in Graves' disease. Clin Immunol. (2022) 245:109160. doi: 10.1016/j.clim.2022.109160 36270470

[34] Douglas RS Ugradar S Mester T Parunakian E Smith TJ . Thyroid eye disease (TED) monocytes express a functional thyrotropin receptor and express increased IL-6 and IL-8: possible mechanistic implications. Ophthalmic Plast Reconstr Surg. (2026) 42:463–72. doi: 10.1097/iop.0000000000003158 41687076

[35] Zhao J Liu X Xu J Fang Y Du P Gao C . Elevated expression and activation of GPR15 in immune cells in Graves' disease. Biomolecules. (2022) 12:1899. doi: 10.3390/biom12121899 36551327 PMC9776225

[36] Wang D Ling J Tan RQ Wang H Qu Y Li X . CD169+ classical monocyte as an important participant in Graves' ophthalmopathy through CXCL12-CXCR4 axis. iScience. (2024) 27:109213. doi: 10.1016/j.isci.2024.109213 38439953 PMC10910260

[37] van Steensel L Paridaens D van Meurs M van Hagen PM van den Bosch WA Kuijpers RW . Orbit-infiltrating mast cells, monocytes, and macrophages produce PDGF isoforms that orchestrate orbital fibroblast activation in Graves' ophthalmopathy. J Clin Endocrinol Metab. (2012) 97:E400–8. doi: 10.1210/jc.2011-2697 22238384

[38] Zeiträg J Benedicic M Wolf J Ammon T Mayr V Holthoff HP . Inflammatory and tolerogenic dendritic cells and T lymphocytes in Graves' thyroidal and orbital disease. Biochim Biophys Acta Mol Basis Dis. (2025) 1871:167747. doi: 10.1016/j.bbadis.2025.167747 40024060

[39] Tiucă OM Morariu SH Mariean CR Tiucă RA Nicolescu AC Cotoi OS . Predictive performances of blood-count-derived inflammatory markers for liver fibrosis severity in psoriasis vulgaris. Int J Mol Sci. (2023) 24:16898. doi: 10.3390/ijms242316898 38069218 PMC10707279

[40] Yang Y Huang W He X Qu X Cai J Zhou F . Association between monocyte-to-lymphocyte ratio and metabolic dysfunction-associated steatotic liver disease and hepatic steatosis: evidence from NHANES 2017–2020. Transl Gastroenterol Hepatol. (2026) 11:7. doi: 10.21037/tgh-25-110 41675314 PMC12887351

[41] Yorke E . Hyperthyroidism and liver dysfunction: a review of a common comorbidity. Clin Med Insights Endocrinol Diabetes. (2022) 15:11795514221074672. doi: 10.1177/11795514221074672 35153522 PMC8829710

[42] Song P Zhang J Zhang Y Shu Z Xu P He L . Hepatic recruitment of CD11b+Ly6C+ inflammatory monocytes promotes hepatic ischemia/reperfusion injury. Int J Mol Med. (2018) 41:935–45. doi: 10.3892/ijmm.2017.3315 29251315 PMC5752159

[43] Dong X Liu J Xu Y Cao H . Role of macrophages in experimental liver injury and repair in mice. Exp Ther Med. (2019) 17:3835–47. doi: 10.3892/etm.2019.7450 31007731 PMC6468932

[44] He K Hu Y Xu XH Mao XM Hu Y Hu Y . Hepatic dysfunction related to thyrotropin receptor antibody in patients with Graves' disease. Exp Clin Endocrinol Diabetes. (2014) 122:368–72. doi: 10.1055/s-0034-1375667 24941434

[45] Zhang W Tian LM Han Y Ma HY Wang LC Guo J . Presence of thyrotropin receptor in hepatocytes: not a case of illegitimate transcription. J Cell Mol Med. (2009) 13:4636–42. doi: 10.1111/j.1582-4934.2008.00670.x 19187127 PMC4515077

[46] Li J Sun X Yao D Xia J . Elevated serum IL-17 expression at cessation associated with Graves’ disease relapse. Int J Endocrinol. (2018) 2018:5689030. doi: 10.1155/2018/5689030 29713343 PMC5866853

[47] Torimoto K Okada Y Nakayamada S Kubo S Kurozumi A Narisawa M . Comprehensive immunophenotypic analysis reveals the pathological involvement of Th17 cells in Graves' disease. Sci Rep. (2022) 12:16880. doi: 10.1038/s41598-022-19556-z 36207336 PMC9546934

[48] Hashimoto K Nishihara E Matsumoto M Matsumoto S Nakajima Y Tsujimoto K . Sialic acid-binding immunoglobulin-like lectin1 as a novel predictive biomarker for relapse in Graves' disease: a multicenter study. Thyroid. (2018) 28:50–9. doi: 10.1089/thy.2017.0244 29037117

[49] Gu P Xu P Chen Y Li J Sun H Xu H . The predictive value of pan-immune inflammatory index for early recurrence of atrial fibrillation after cryoablation. BMC Cardiovasc Disord. (2024) 24:669. doi: 10.1186/s12872-024-04329-5 39580428 PMC11585142

[50] Liu B Liu D Qi C Sunggip C . Pan-immune-inflammation value and systemic immune-inflammation index predict the clinical efficacy of biological agents in Crohn's disease. Postgrad Med J. (2026) 102:378–84. doi: 10.1093/postmj/qgaf172 41092350

[51] Kim M Kim BH Jang MH Kim JM Kim EH Jeon YK . High neutrophil-to-lymphocyte ratio is associated with relapse in Graves' disease after antithyroid drug therapy. Endocrine. (2020) 67:406–11. doi: 10.1007/s12020-019-02137-y 31749116

