# Supplementary material for: Peripheral blood inflammatory indices across thyroid functional states in Graves’ disease: associations with liver biochemical abnormalities and hyperthyroid relapse
Source: Front Endocrinol (Lausanne). 2026 Aug 21;17:1863070. doi: 10.3389/fendo.2026.1863070 (PMC13541543; doi:10.3389/fendo.2026.1863070)
Supplement: Supplementary file 1 [file Table1.docx]

**Supplementary Table S1. Laboratory reference intervals for thyroid function and liver biochemical parameters.**

| **Category** | **Parameter** | **Reference interval** |
| --- | --- | --- |
| Thyroid function | TT3 | 1.01–2.96 nmol/L |
|  | TT4 | 55.40–161.25 nmol/L |
|  | FT3 | 2.77–6.31 pmol/L |
|  | FT4 | 10.44–24.38 pmol/L |
|  | TSH | 0.380–4.340 μIU/mL |
|  | TRAb | 0–1.5 IU/L |
| Liver biochemical parameters | TBIL | 0–21 μmol/L |
|  | DBIL | 0–4 μmol/L |
|  | IBIL | 0–17 μmol/L |
|  | ALT | 0–40 U/L |
|  | AST | 0–35 U/L |

**Notes:** Reference intervals were those used by the central clinical laboratory of Lanzhou University Second Hospital during the study period. TT3, total triiodothyronine; TT4, total thyroxine; FT3, free triiodothyronine; FT4, free thyroxine; TSH, thyroid-stimulating hormone; TRAb, thyrotropin receptor antibody; TBIL, total bilirubin; DBIL, direct bilirubin; IBIL, indirect bilirubin; ALT, alanine aminotransferase; AST, aspartate aminotransferase.

Supplementary Table S2. Baseline characteristics of patients in the OH group at different treatment stages.

| **Variable** | **OH-IP (n = 54)** | **OH-ATD (n = 191)** | **OH-RP (n = 38)** | **H/χ²/F** | ***P* value** |
| --- | --- | --- | --- | --- | --- |
| Age (years) | 38.50 (26.75, 50.25) | 36.00 (29.00, 47.00) | 39.50 (30.50, 50.25) | 2.024 | 0.364 |
| Male, n (%) | 16 (29.63%) | 68 (35.60%) | 13 (34.21%) | 0.667 | 0.717 |
| TT3 (nmol/L) | 4.77 (3.12, 9.65) | 2.61 (2.11, 3.40)^**^ | 3.17 (2.57, 4.14)^*#^ | 41.489 | **<0.001** |
| TT4 (nmol/L) | 259.00 (211.60, 387.00) | 169.65 (135.45, 204.18)^**^ | 208.15 (154.68, 252.98)^**#^ | 42.683 | **<0.001** |
| TSH (μIU/mL) | 0.006 (0.003, 0.009) | 0.007 (0.005, 0.013)^**^ | 0.008 (0.005, 0.012) | 7.342 | **0.025** |
| TRAb (IU/L) | 13.10 (5.53, 28.13) | 8.73 (2.82, 22.80) | 9.80 (3.19, 27.33) | 4.398 | 0.111 |
| FT3 (pmol/L) | 22.10 (12.18, 30.80) | 8.99 (7.14, 13.64)^**^ | 9.51 (7.23, 17.36)^**^ | 48.558 | **<0.001** |
| FT4 (pmol/L) | 54.48 (37.85, 100.00) | 25.98 (21.24, 34.78)^**^ | 31.34 (21.48, 50.44)^**^ | 58.336 | **<0.001** |
| WBC (×10⁹/L) | 5.63 (4.85, 7.42) | 5.95 (4.87, 6.99) | 6.06 (5.45, 7.27) | 1.020 | 0.601 |
| PDW (%) | 13.78 (10.80, 16.10) | 15.10 (11.00, 16.20) | 14.10 (10.70, 16.15) | 0.276 | 0.871 |
| MPV (fL) | 10.10 (9.78, 10.80) | 10.00 (9.40, 10.80) | 10.55 (9.68, 11.80) | 5.424 | 0.066 |
| PCT (%) | 0.27 (0.24, 0.31) | 0.25 (0.22, 0.28)^*^ | 0.28 (0.23, 0.31)^#^ | 9.229 | **0.010** |
| P-LCR (%) | 26.50 (23.75, 31.00) | 26.00 (21.00, 31.00) | 30.50 (22.75, 40.00) | 4.982 | 0.083 |
| NEUT (×10⁹/L) | 2.92 (2.00, 3.90) | 3.21 (2.27, 4.10) | 3.61 (3.01, 4.34)^**#^ | 7.291 | **0.026** |
| LYM (×10⁹/L) | 2.26 (1.63, 2.77) | 2.01 (1.71, 2.39) | 1.83 (1.41, 2.22)^**#^ | 9.363 | **0.009** |
| MONO (×10⁹/L) | 0.49 (0.40, 0.60) | 0.40 (0.32, 0.52)^**^ | 0.52 (0.39, 0.62)^##^ | 21.733 | **<0.001** |
| Hb (g/L) | 147.31 ± 17.05 | 149.34 ± 18.62 | 148.66 ± 19.24 | 0.250 | 0.773 |
| PLT (×10⁹/L) | 267.69 ± 71.52 | 249.73 ± 58.53 | 255.61 ± 62.66 | 1.797 | 0.168 |
| ALT (U/L) | 26.50 (19.25, 33.00) | 21.00 (15.00, 32.00) | 21.00 (16.75, 28.25) | 4.851 | 0.088 |
| AST (U/L) | 29.00 (24.00, 39.25) | 26.00 (21.00, 34.25) | 29.50 (21.00, 35.75) | 1.842 | 0.398 |
| TBIL (μmol/L) | 15.95 (11.43, 20.68) | 14.15 (11.05, 18.73) | 15.45 (11.40, 20.93) | 2.341 | 0.310 |
| DBIL (μmol/L) | 3.60 (2.73, 4.50) | 2.90 (2.10, 4.30) | 3.00 (2.20, 4.53) | 4.299 | 0.117 |
| IBIL (μmol/L) | 12.10 (8.43, 15.68) | 10.75 (8.20, 14.83) | 12.85 (9.10, 16.73) | 2.643 | 0.267 |

Notes: Data are presented as mean ± SD, median (Q1, Q3), or n (%), as appropriate. **P* < 0.05 and ***P* < 0.01 versus the OH-IP group; #*P* < 0.05 and ##*P* < 0.01 for the comparison between the OH-ATD and OH-RP groups. Bold *P* values indicate statistical significance at *P* < 0.05.

**Supplementary Table S3. Baseline characteristics of patients in the EU group at different treatment stages.**

| **Variable** | **EU-ATD (n = 170)** | **EU-WD (n = 97)** | **H/χ²/F** | ***P* value** |
| --- | --- | --- | --- | --- |
| Age (years) | 37.00 (29.00, 49.00) | 38.00 (33.50, 50.50) | 3.516 | 0.061 |
| Male, n (%) | 37 (21.77%) | 20 (20.62%) | 0.048 | 0.826 |
| TT3 (nmol/L) | 1.57 ± 0.28 | 1.60 ± 0.29 | 0.691 | 0.407 |
| TT4 (nmol/L) | 105.64 ± 23.51 | 113.82 ± 23.41 | 4.591 | **0.034** |
| TSH (μIU/mL) | 1.97 (0.98, 3.00) | 1.93 (1.18, 2.70) | 0.047 | 0.828 |
| TRAb (IU/L) | 2.22 (1.56, 4.33) | 1.40 (0.69, 1.80) | 54.867 | **<0.001** |
| FT3 (pmol/L) | 5.02 (4.58, 5.46) | 5.17 (4.71, 5.57) | 3.051 | 0.081 |
| FT4 (pmol/L) | 15.37 ± 2.67 | 16.31 ± 2.41 | 8.072 | **0.005** |
| WBC (×10⁹/L) | 6.14 (5.05, 7.18) | 5.48 (4.77, 6.37) | 6.200 | **0.013** |
| PDW (%) | 13.40 (10.88, 16.10) | 13.50 (11.45, 16.30) | 1.730 | 0.188 |
| MPV (fL) | 10.20 (9.50, 10.90) | 10.20 (9.80, 11.00) | 0.542 | 0.462 |
| PCT (%) | 0.25 (0.21, 0.29) | 0.23 (0.21, 0.27) | 2.824 | 0.093 |
| P-LCR (%) | 26.00 (21.00, 32.25) | 27.00 (23.50, 32.50) | 0.545 | 0.460 |
| NEUT (×10⁹/L) | 3.46 (2.81, 4.30) | 3.09 (2.39, 3.53) | 9.974 | **0.002** |
| LYM (×10⁹/L) | 2.01 (1.66, 2.41) | 2.09 (1.67, 2.44) | 0.387 | 0.534 |
| MONO (×10⁹/L) | 0.35 (0.30, 0.43) | 0.34 (0.29, 0.41) | 0.814 | 0.367 |
| Hb (g/L) | 145.59 ± 16.73 | 146.11 ± 17.28 | 0.059 | 0.808 |
| PLT (×10⁹/L) | 246.95 ± 60.97 | 237.28 ± 58.53 | 1.599 | 0.207 |
| ALT (U/L) | 14.00 (10.00, 19.50) | 13.00 (9.00, 17.75) | 1.092 | 0.296 |
| AST (U/L) | 24.00 (19.00, 29.00) | 21.00 (19.00, 28.75) | 1.253 | 0.263 |
| TBIL (μmol/L) | 13.00 (10.00, 17.30) | 13.20 (11.10, 16.10) | 0.033 | 0.856 |
| DBIL (μmol/L) | 2.70 (1.85, 3.75) | 2.45 (1.50, 3.15) | 3.643 | 0.056 |
| IBIL (μmol/L) | 10.40 (7.35, 13.75) | 10.70 (8.78, 13.43) | 0.572 | 0.450 |

Notes: Data are presented as mean ± SD, median (Q1, Q3), or n (%), as appropriate. Bold *P* values indicate statistical significance at *P* < 0.05.

**Supplementary Table S4. Collinearity diagnostics for the full multivariable model and final prediction models.**

| **Analysis/model** | **Variable** | **Tolerance** | **VIF** |
| --- | --- | --- | --- |
| Full multivariable model for liver biochemical abnormalities | Sex | 0.547 | 1.827 |
|  | Treatment status | 0.847 | 1.181 |
|  | TRAb | 0.938 | 1.066 |
|  | FT4 | 0.811 | 1.234 |
|  | Hb | 0.550 | 1.818 |
|  | ZMLR | 0.915 | 1.093 |
| TRAb–Hb–ZMLR prediction model for liver biochemical abnormalities | TRAb | 0.996 | 1.004 |
|  | Hb | 0.997 | 1.003 |
|  | ZMLR | 0.995 | 1.005 |
| TRAb–PIV prediction model for hyperthyroid relapse | TRAb | 0.997 | 1.003 |
|  | PIV | 0.997 | 1.003 |

**Notes:** Multicollinearity was assessed among all predictors simultaneously included in the full multivariable model and in each final combined prediction model. Tolerance ≤ 0.20 or VIF ≥ 5 was considered indicative of potentially problematic multicollinearity. No variable met either threshold.
